# Diffuse reflectance spectroscopy (DRS) and infrared (IR) measurements for studying biofilm formation on common plastic litter polymer (LDPE and PET) surfaces in three different laboratory aquatic environments

**DOI:** 10.1007/s11356-023-27163-2

**Published:** 2023-04-28

**Authors:** Pavlos Tziourrou, John Vakros, Hrissi Kassiani Karapanagioti

**Affiliations:** 1 Department of Chemistry, University of Patras, 26504 Patras, Greece; 2 School of Sciences and Engineering, University of Nicosia, 2417 Nicosia, Cyprus

**Keywords:** Biofilm formation, Bioreactors, Keto index, Marine debris, Spectra, Surface functional groups

## Abstract

Different species of microorganisms colonize the plastic surfaces and form biofilms depending on the aquatic environment. In the current investigation, characteristics of the plastic surface after exposure to three different aquatic environments based on visualization using scanning electron microscopy (SEM) and spectroscopic (diffuse reflectance (DR) and infrared (IR)) techniques were examined in laboratory bioreactors with time. For both materials, there were no differences observed in the ultraviolet (UV) region among the reactors and several peaks were observed with fluctuating intensities and without any trends. For light density polyethylene (LDPE), peaks indicating the presence of biofilm could be observed in the visible region for activated sludge bioreactor, and for polyethylene terephthalate (PET), freshwater algae biofilm was also visible. PET in freshwater bioreactor is the most densely populated sample both under the optical microscope and SEM. Based on the DR spectra, different visible peaks for LDPE and PET were observed but, in both cases, the visible region peaks (~ 450 and 670 nm) correspond to the peaks found in the water samples of the bioreactors. The difference on these surfaces could not be identified with IR but the fluctuations observed in the UV wavelength region were also detectable using indices obtained from the IR spectra such as keto, ester, and vinyl. For instance, the virgin PET sample shows higher values in all the indices than the virgin LDPE sample [(virgin LDPE: ester Index (I) = 0.051, keto I = 0.039, vinyl I = 0.067), (virgin PET: ester I = 3.5, keto I = 19, vinyl I = 0.18)]. This suggests that virgin PET surface is hydrophilic as expected. At the same time, for all the LDPE samples, all the indices demonstrated higher values (especially for R2) than the virgin LDPE. On the other hand, ester and keto indices for PET samples demonstrated lower values than virgin PET. In addition, DRS technique was able to identify the formation of the biofilm both on wet and dry samples. Both DRS and IR can describe changes in the hydrophobicity during the initial formation of biofilm but DRS can better describe the fluctuations of biofilm in the visible spectra region.

**Supplementary Information:**

The online version contains supplementary material available at 10.1007/s11356-023-27163-2.

## Introduction

There is a relative high number of studies on environmental burden caused by plastic litter (Takada and Karapanagioti 2019; Karapanagioti and Kalavrouziotis 2019). The most widely spread plastic waste are low-density polyethylene (LDPE, LLDPE), high-density polyethylene (HDPE), polypropylene (PP), polystyrene (PS), foamed polystyrene, nylon (PA), polyethylene terephthalate (PET), poly(vinyl chloride) (PVC), and cellulose acetate (CA) (Andrady et al. 2011). They can be observed in different aquatic environments, such as marine environment (Schneider et al. 2018), lakes, rivers, as well as urban areas from which they end up in artificial aquatic environments, i.e., wastewater treatment plants (Blettler et al. 2018). According to a review study (Schwarz et al. 2019) about different plastic types in aquatic environments, a relative high input of PET was mentioned (density class: high), while polyethylene (PE) was detected in all aquatic fields.

A film of microbial diversity called biofilm (Donlan 2002), or plastishere in the case of plastic surface (Rummel et al. 2017; Zettler et al. 2013) is potentially developed on any surface in an aqueous environment. In a recent study by Tziourrou et al. (2020), using diffuse reflectance spectroscopy (DRS) technique, they observed that biofilm in the marine environment does not develop randomly but in a specific way, depending on the type of plastic material. The steps followed by the creation and development of a biofilm on a surface are generally as follows: (a) reversible attachment of bacteria, (b) irreversible attachment of a surface by microorganisms, (c) biofilm maturation, and (d) biofilm dispersion (Khatoon et al. 2018; Donlan 2002). Studies have reported chemical alterations (i.e., functional groups) of plastics due to their exposure to microorganisms [Fotopoulou and Karapanagioti (2017) and references within].

The techniques used to study biofilms can vary from biological, physicochemical (Azeredo et al. 2017), microscopy techniques (Pantanella et al. 2013) to mathematical modeling (Wilson et al. 2017). There are very few publications which study biofilm on plastics directly after sampling from the field or during a laboratory experiment, without any pretreatment or alteration of the sample (e.g., Tziourrou et al. 2020; Sfaelou et al. 2015a); in other words, the study of biofilm as a material that alters polymer surfaces at different periods (e.g., color change of biofilm) needs to be further studied.

UV-visible (UV-vis) spectroscopy can be used as a useful method for microorganism determination (Badri et al. 2015). According to Alupoaei and Garcia-Rubio (2005), UV-vis spectra of microorganisms contain information on their properties (e.g., chemical composition, internal structure, number, size, and shape). Correlating natural and artificial marine environments, the research supported that biofilm patterns were related to DRS peaks; more specifically, the fluctuation of the DR spectra depended on the polymer type and the time period of biofilm formation on plastic surfaces (Tziourrou et al. 2020).

The purpose of the present study was to compare the biofilm formation on two commonly used plastics such as LDPE and PET in three different laboratory aqueous systems with various microorganisms. Specifically, the laboratory bioreactors were used to simulate a marine environment, the aeration tank of a wastewater treatment plant, and a freshwater pond environment. At different residence times, biofilms were characterized (using DRS) in both materials and simultaneous check for changes in their IR spectra. The specific objectives of the present study were (a) to check if the DRS measurements would provide different spectra for biofilm formed by different microorganisms and on various plastic surfaces; (b) to identify changes in the spectra due to the desiccation of samples, e.g., dry plastic samples collected from a beach; (c) to compare information collected with DRS to that collected with IR spectra and determine their qualitative differences.

The main scope of this work is to demonstrate that DRS is a valuable technique, very fast and with low cost, and that it can be applied to give information about the formation of biofilm even in almost not-detectable conditions. Also, it can give this information independably of the biofilm type, bacteria type, and time. It may be used for the identification of the biofilm on polymer surfaces. The proposed technique can be directly applied in the field, demands no special treatment, and even no highly qualified personnel, it is not destructive, which is very important in laboratory experiments, in food industry and in many more applications.

Polyethylene is the most common polymer produced and thus, the most found in the environment. LDPE and PET were chosen in the present study because they are very different in the polarity and the surface groups. It is considered important to investigate if the polarity of the support (polymer) can alter the macro-characteristics and the amount of the biofilm. This study involves many different parameters such as 2 polymers, 3 bioreactors, 2 different analytical techniques, 2 different sample states, dry and wet, and several different sampling points in time.

## Materials and methods

### Bioreactor setup and operation

**Fig. 1:**
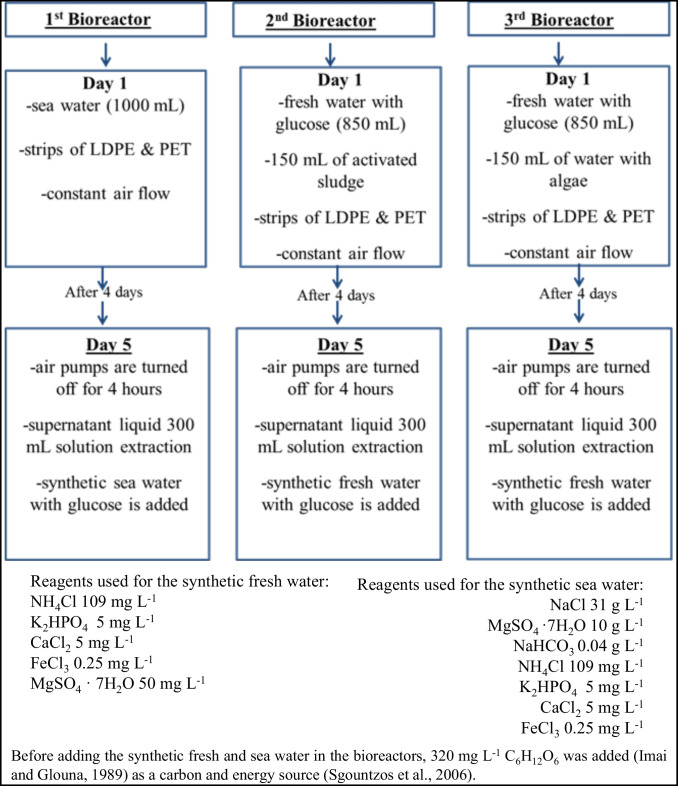
Flow diagram of the steps taken during the experiment

Three glass batch bioreactor systems (1.5 L at 25 ± 2 °C) were developed for the experiment purposes (Fig. S1) (Sfaelou et al. 2016). Each reactor was operated in subsequent cycles of 5 days [more specifically a 4-day operation of bioreactors plus one day of refreshing the liquids based on Tziourrou et al. (2020) and the energy source (C_6_H_12_O_6_) (Imai and Gloyna 1990; Sgountzos et al. 2006)]; a similar procedure was followed by Sfaelou et al. (2015a, b, 2016) and Papadimitriou et al. (2010, 2018)]. There was constant air flow via air pumps in each reactor (dissolved oxygen is consumed by the microorganisms as an electron acceptor during aerobic growth). The total solution volume in each reactor was 1L (Sfaelou et al. 2015a). In Fig. 1, a flow diagram of the steps taken during the experiment is illustrated.

The above process (Fig. 1) was repeated for 50 days and based on our previous study (Tziourrou et al. 2020), measurements were taken on the 1st, 2nd, 5th, 13th, 18th, 33rd, 40th, 45th, and 50th days from the starting day.

### Visualization techniques

The visualization of biofilm from R1, R2, and R3 by optical microscope Leica DMLB (Leica, Germany) was carried out only on PET surfaces, due to the transparent nature of the material. For the visualization of biofilm on both plastic materials in different magnifications, scanning electron microscope (SEM) JSM 6300 of the company JEOL was used. For selected samples, X-ray energy-dispersive spectrometer (EDS) after coating by C was used for surface elemental analyses. For all optical observations, day 33 was chosen. The presence of microorganisms were visualized with a FEI QUANTA 250 FEG (SEM), with FE source and analysis under 10 to 4000 Pa pressure.

### Spectroscopic characterization of the samples

UV-vis spectrophotometer (Varian Cary 3) equipped with an integration sphere for the DRS measurements was used. The absorption spectra of the liquid phase of the bioreactors were recorded from 200 to 800 nm. Subsequently, for the comparison of the initial and final stages (namely after creating biofilm) spectra were obtained in the UV-vis region (ultraviolet-visible) using DRS (diffuse reflection UV-vis spectroscopy) method. Each time, a virgin plastic strip by the same material was used as blank against plastic strip with biofilm; thus, peaks of DR spectra were related to the biofilm formed on the plastic strips.

The Kubelka-Munk equation (outcome is F(R)) is used for DR spectroscopy. For the solid samples the technique measures the reflectance of the visible radiation spectrum that cannot penetrate the sample. The F(R) which is an expression taking into account the absorption and scattering that result from the sample, rather than the reflectance, can be used. These values are proportional to the absorbance of the sample. This is a method used in the past by our group for the observation of biofilm formation (Tziourrou et al. 2020; Sfaelou et al. 2015a) and polymer degradation (Rtimi et al. 2015).

For functional groups identification for these samples, attenuated total reflectance–Fourier transform infrared spectroscopy (ATR-FTIR) (model: “Bruker Optics’ Alpha-P Diamond ATR Spectrometer of Bruker Optics GmbH”) was used. The measurement range was 400–4000 cm^−1^ with a resolution of 4 cm^−1^ and 24 scans (Tziourrou et al. 2019). Before the measurements, the samples were air-dried, and biofilm was not removed from the plastic surfaces.

## Results

### Visualization of the plastic surfaces

**Fig. 2:**
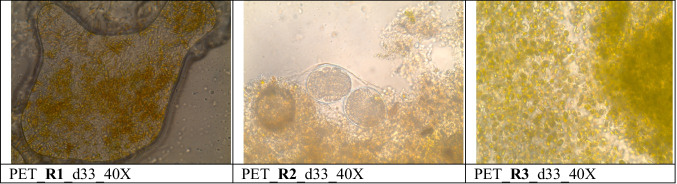
Optical microscope images (magnification 40×) of biofilm on PET surfaces (R1, R2, and R3) on day 33. The images were taken directly on the transparent plastic

**Fig. 3:**
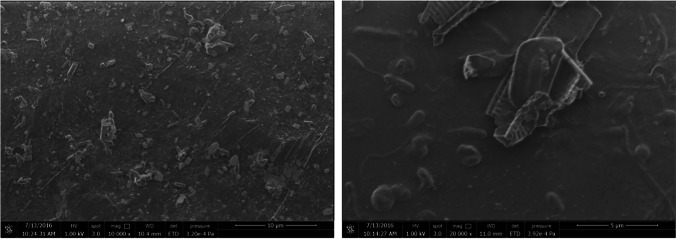
SEM images of LDPE (left) and PET (right) surfaces were in R1 for 33 days

**Fig. 4:**
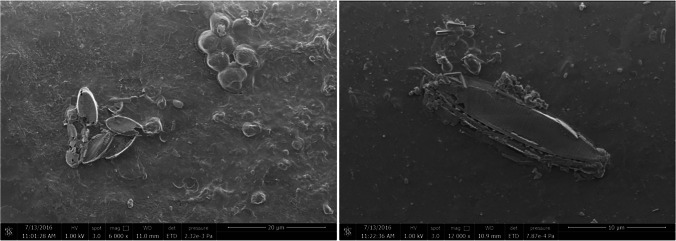
SEM images of LDPE (left) and PET (right) surfaces were in R2 for 33 days

**Fig. 5:**
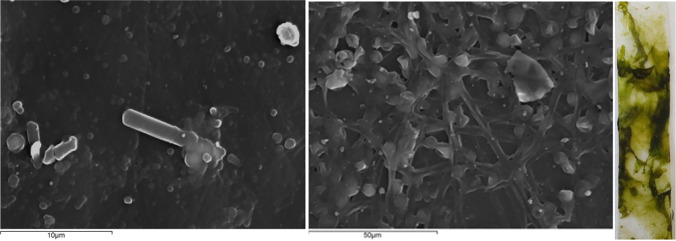
SEM images of LDPE (left) and PET (middle) surfaces were in R3 for 33 days. Photograph (*macroscopic observation*) of the PET plastic strip with biofilm (45th day) (right)

Based on optical microscope images, on each PET surface in the bioreactors, microorganism species of different shape, color, size, and abundance were observed (Fig. 2). Differences of LDPE surfaces (SEM images) in R1 (Fig. 3), R2 (Figs. 4 and S2), and R3 (Figs. 5 and S3) were also observed. The sample from R2 seems to be the most diverse in terms of the number of species (Figs. 4 and S2). EDS analysis indicate silicon (Si) on the sample, which is the element of the shell of diatoms (Dugdale and Wilkerson 2001) (Fig. S2). Based on the optical microscope image for PET from R2, a possible common (with circular shape) microorganism can be observed in correlation with the SEM image for LDPE from R2. Shells of microorganisms (diatoms) were observed on PET surfaces both for R1 and R2. For R3, algae were mainly observed on PET surface (Fig. 5).

### UV-vis interpretation

**Fig. 6:**
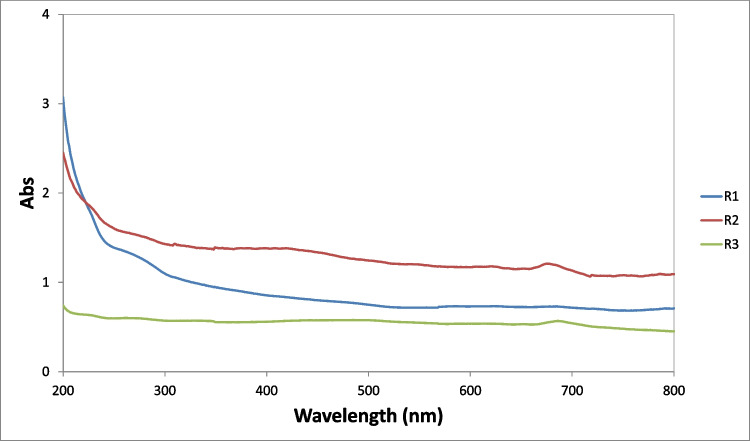
Absorption (Abs) spectra of each bioreactor liquid. Blue line corresponds to R1 with synthetic seawater and microorganisms from marine environment, red line corresponds to R2 (synthetic fresh water and microorganisms from activated sludge), and green line corresponds to R3 (synthetic fresh water with green algae)

Absorption (Abs) spectra of the liquid phase from the three bioreactors are presented in Fig. 6. In Abs spectra, each bioreactor sample demonstrated shoulders as follows: at 275 nm for R1, R2, and R3, at 420 nm for R2, at 225 nm and 330 nm for R3 and peaks at 675 nm for R2, and 687 nm for R3. A similar spectrum for R1 was also recorded by Tziourrou et al. (2020) at an early day and R1 was characterized as low in biodiversity and the number of microorganisms compared to the marine environment. Here, at a later day, only one peak with low intensity was observed since only glucose was used as a carbon source, certain species may grow in favor of others. The other two reactors seem to maintain more microorganisms. The light absorption of the suspensions of R2 and R3 in the visible region (400–800 nm) can be attributed to the colors of the suspensions. In general, the observed spectra were influenced by many parameters such as cell size, internal structure, and chemical composition (Spear et al. 2009).

### DR spectra observations

Different patterns among the three reactors were captured. Selected DR spectra of LDPE and PET from R1, R2, and R3 are illustrated in Figs. 7, and 8 (wet samples) and Figs. 9, and 10 (dry samples), respectively. The complete set of spectra can be found in the Supplementary Materials (Figs. S4–S7).

**Fig. 7:**
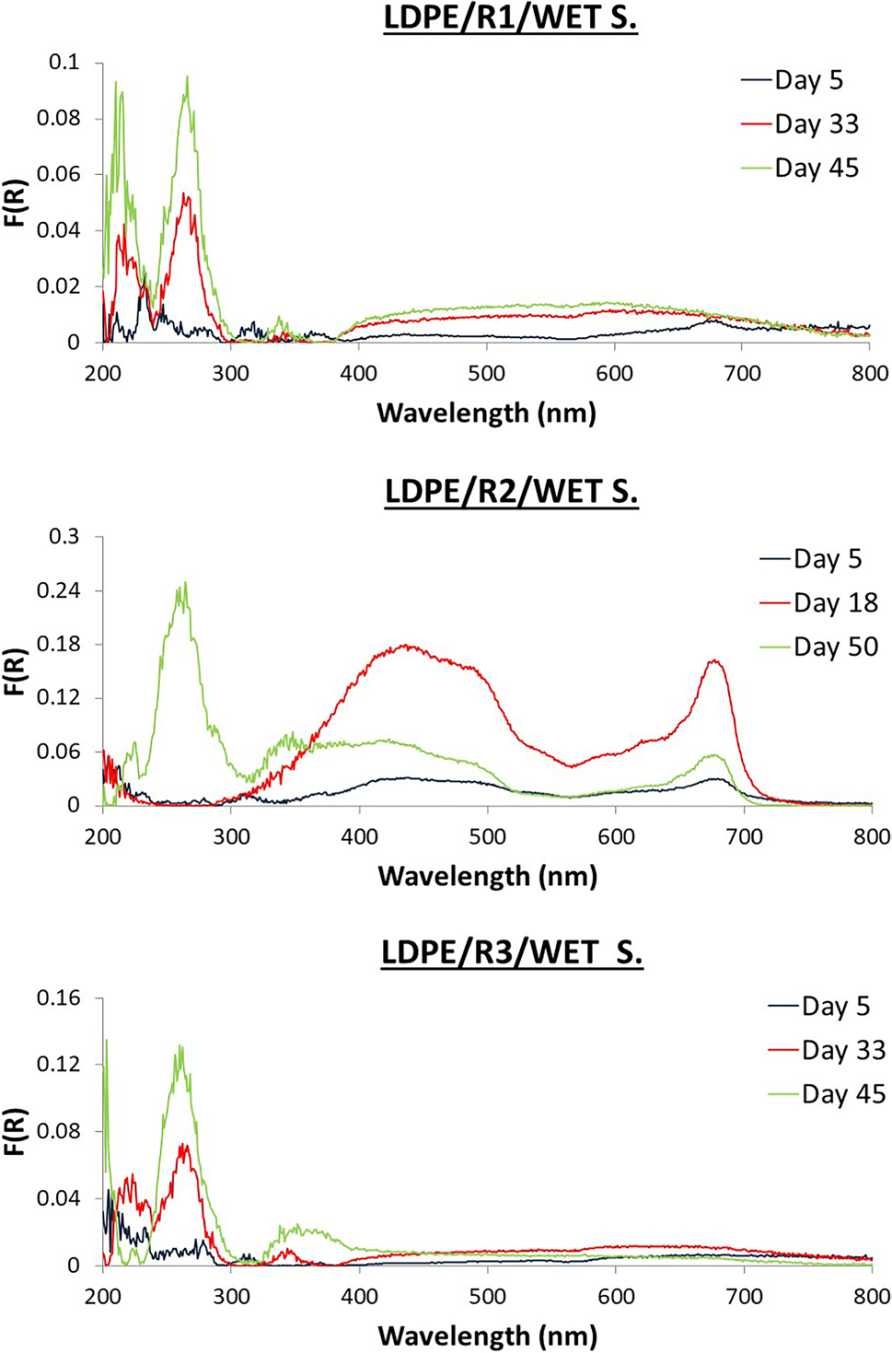
DR spectra of wet biofilm on LDPE from R1, R2, and R3

**Fig. 8:**
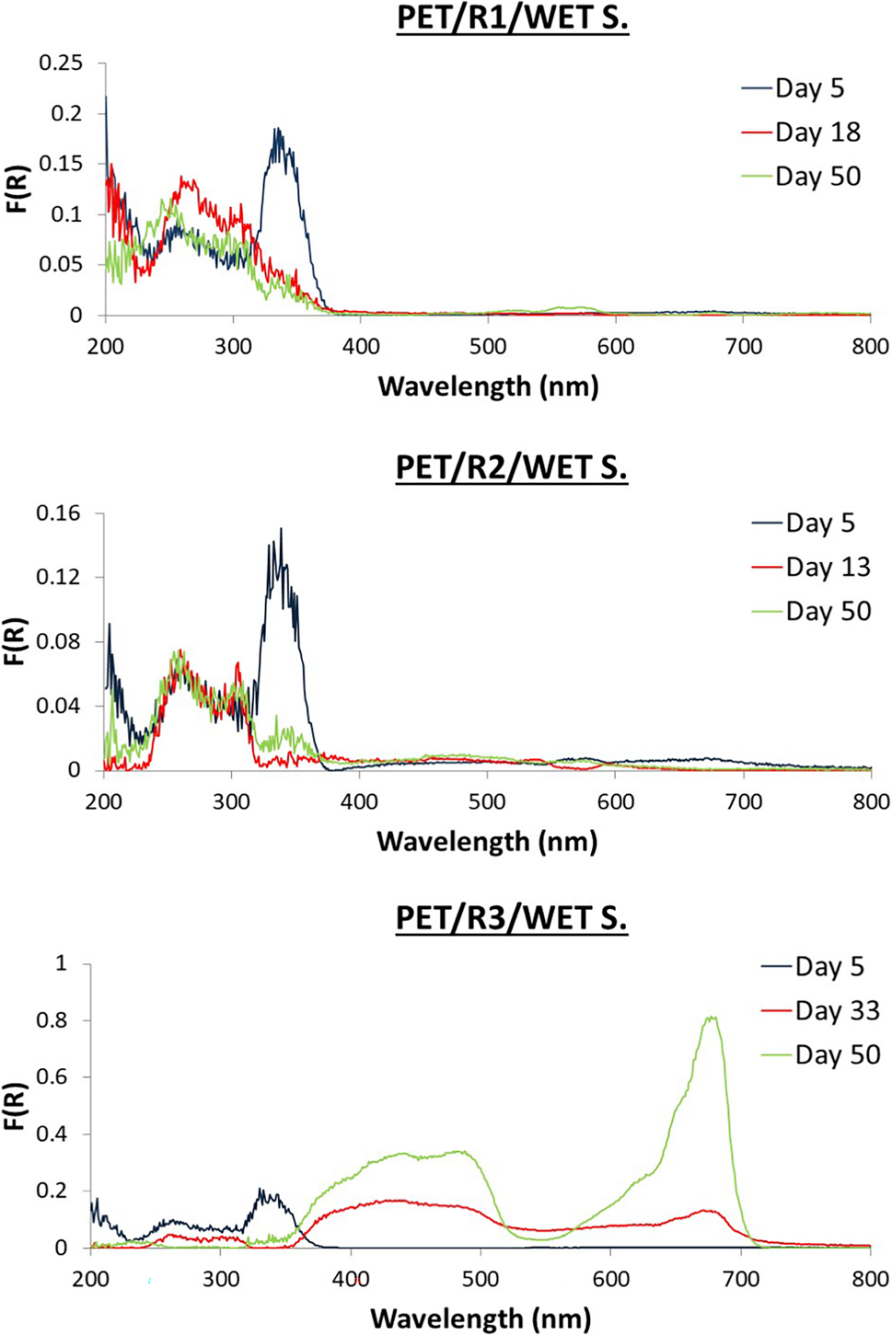
DR spectra of wet biofilm on PET from R1, R2, and R3

**Fig. 9:**
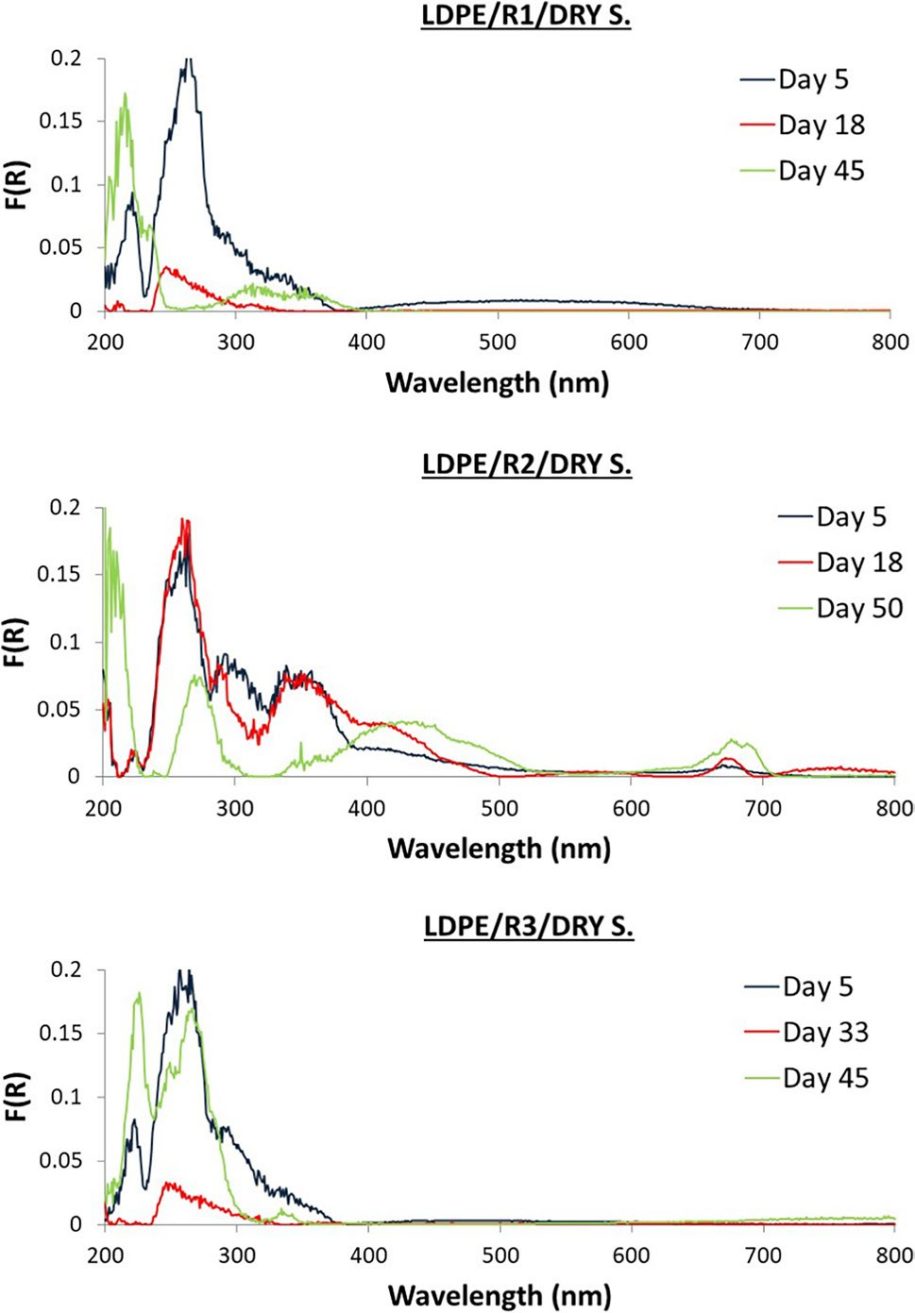
DR spectra of dry biofilm on LDPE from R1, R2, and R3

**Fig. 10:**
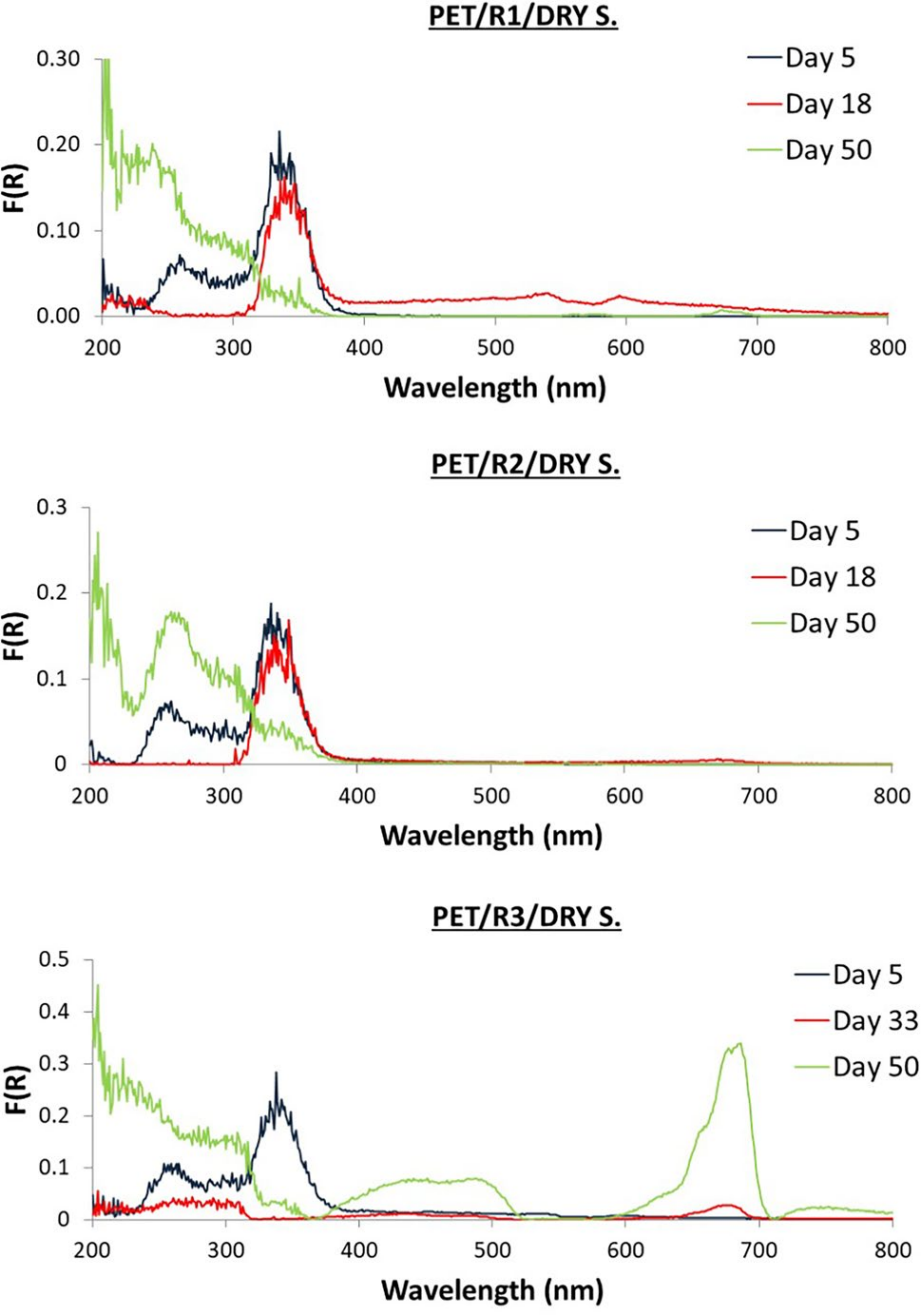
DR spectra of dry biofilm on PET from R1, R2, and R3

### Wet samples from R1

For LDPE, low F(R) value peaks were observed at 200–300 nm. The peaks at 200–300 nm demonstrated higher F(R) values from day 18 to day 45. For PET, 3 main peaks at 200–400 were observed with fluctuating values throughout the whole experiment.

### Dry samples from R1

No significant qualitative differences between LDPE dry and wet samples were observed. However, for each of the days presented, it seems that one of the two peaks becomes more dominant and the values for dry are higher on days 5 and 45. For PET dry samples, similar peaks (200–400 nm) to wet samples were observed. F(R) values of dry PET samples (~ 0.35) are higher than of wet PET samples (~ 0.15). Also, one of the peaks becomes more dominant.

### Wet samples from R2

For LDPE, peaks started with low F(R) values on days 1 and 2 at 200–400 nm. On day 5, a shoulder at 400–700 nm appeared, creating two peaks at 450 and 670 nm on day 13 until day 50. For PET, from day 1 spectrum started from high F(R) value peaks at 200–400 nm. Finally, the peaks at 200–350 nm increased in F(R) value during the last sampling day.

### Dry samples from R2

For LDPE, different intensities in DR spectra were observed compared to wet samples. Peaks with similar wavelengths (200–400 nm) were observed while the peaks at 400–700 nm were also observed with lower intensities compared to wet samples. Peaks for PET fluctuated from 200 to 400 nm with increasing intensities compared to wet PET samples.

### Wet samples from R3

For LDPE, fluctuations of peaks at UV region (200–400 nm) were observed. For PET samples similar F(R) values (300–400 nm) until day 18 were observed, while from day 33 to day 50, peaks were observed in the visible region (400–700 nm).

### Dry samples from R3

For LDPE, similar peaks were observed at 200–300 nm with higher intensities. For PET, peaks were observed in the UV region with increased intensities, while from day 33 to day 50, peaks were observed in the visible region (400–700 nm) with lower intensities compared to the wet PET samples.

Tables S1–S4 summarize all the peaks for wet and dry samples, while the graph in Fig. S8 illustrates the occurrence frequency of peaks for all the wet samples. According to this graph, higher percentage of the peaks were observed in the UV region than were in the visible region in the DR spectra.

### IR spectra observations

**Fig. 11:**
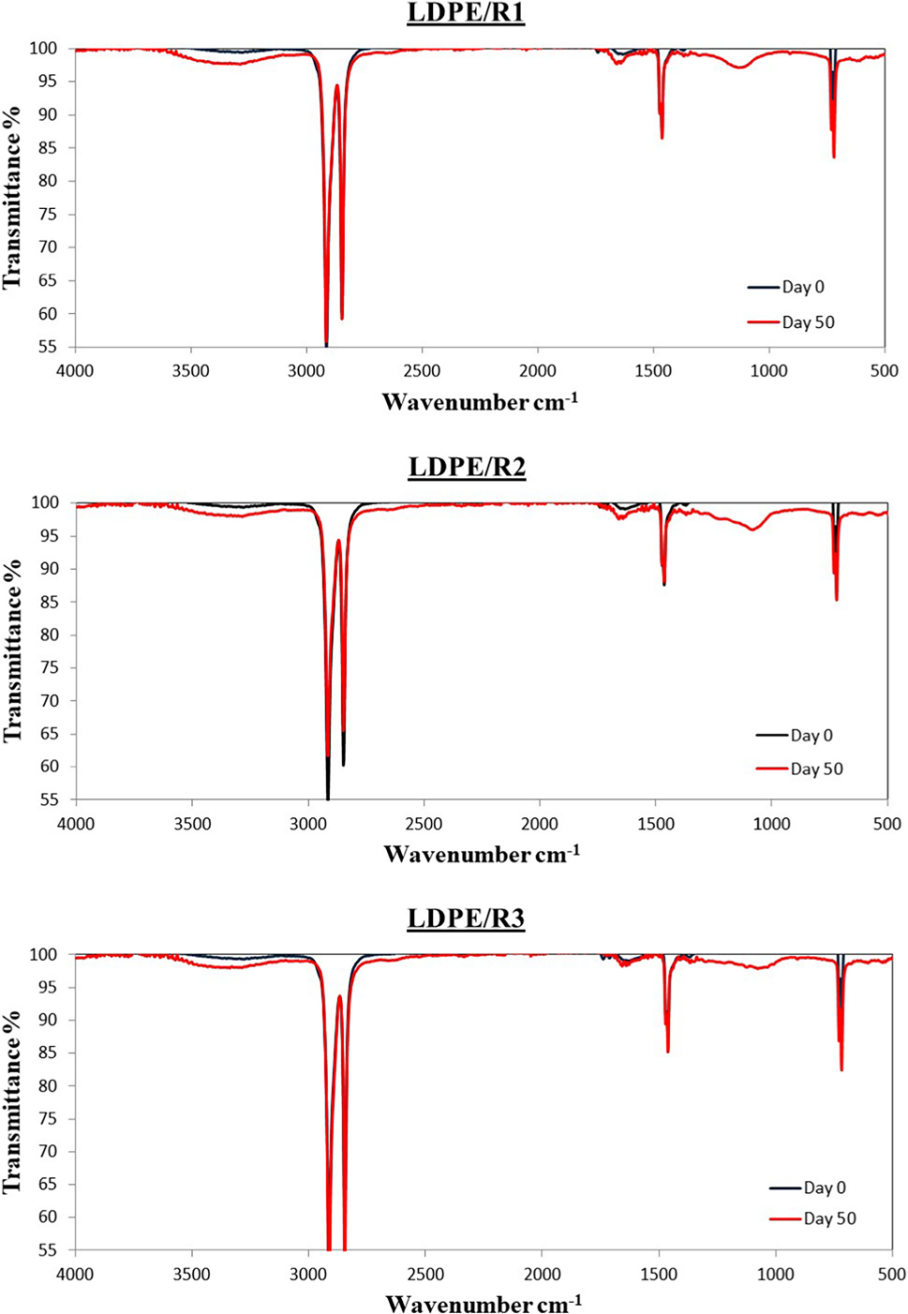
IR spectra of LDPE samples from the three bioreactors

**Fig. 12:**
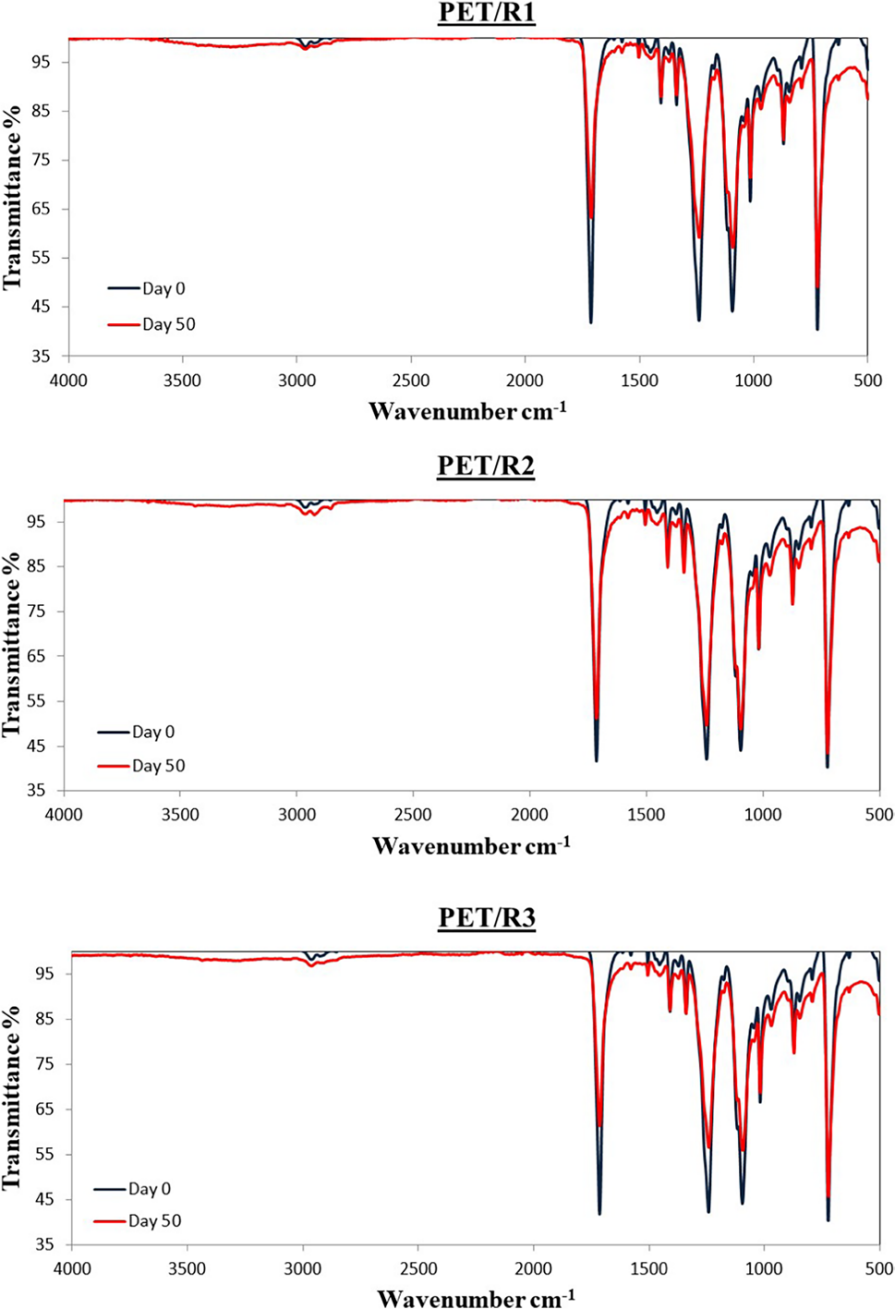
IR spectra of PET samples from the three bioreactors

The ATR-FTIR comparative spectra of the outer surface of the LDPEs and PETs were plotted in a comparative way (Figs. 11 and 12, respectively) for days 0 and 50 whereas the complete set of data are presented in Figs. S10 and S11.

Table S5 shows the extra peaks for the LDPE originating from the three bioreactors (for peaks present in virgin LDPE see Fig. S9). The density and type of these peaks vary greatly mainly from day 5 to day 18 in all three bioreactors. In all three bioreactors, vinyl bond (Albertsson et al. 1987; Harshvardhan and Jha 2013; Fotopoulou and Karapanagioti 2015a) and ester linkage (Fotopoulou and Karapanagioti 2012) were observed. Peak at 1066 cm^−1^ found in R1 and R2 was also referred by Frost et al. (1998) and Guerrero and Maier (2018) who studied dimethyl sulfoxide intercalated kaolinites and barium sulfate, respectively. In R1, C-O stretching of ether group (Gajendiran et al. 2016) at 1081 cm^−1^ from day 5 while 1085 cm^−1^ on day 33 was also observed by Prasad et al. (2011) who studied radiation damage in PET polymer. In R1, C-O stretch (Jung et al. 2018) at 1099 cm^−1^ on day 45 was also observed. Bond at 1544 cm^−1^ in R1 and R2 was also found by Riaz et al. (2018) who investigated natural and synthetic collagen. Finally, 1664 cm^−1^ on day 50 in R1 was observed by Riaz et al. (2018).

For the virgin PET (Fig. S5), five main peaks are identified at wavenumbers 1715, 1245, 1100, 870, and 730 cm^−1^, corresponding in ketones (C = O), ether aromatic (C-O), ether aliphatic (C-O), aromatic (C-H), and aromatic (C-H) bond (Ioakeimidis et al. 2016) (Table S6). For PET with biofilm, similar peaks were observed. Among the native groups of the polymer (PET), there are various peaks that are decreasing, or even disappearing, or fluctuating and are attributed to the biofilm formation on the plastic surface.

## Discussion

### DR spectra interpretation

For both materials, there were no differences observed in the UV region among the reactors and several peaks were observed with fluctuating intensities and without any trends. Plastic type is significant for the first days; biomolecules on PET are attached earlier than on LDPE and are less hydrophobic (Tziourrou et al. 2020) as is PET compared to LDPE (Jones et al. 1999). In general, 𝑛–𝜎∗ and/or 𝜋–𝜋∗ electron transitions (peaks at 200 – 220 nm) can be detected in several organic functional groups while 𝜋–𝜋∗ and *n*–𝜋∗ transitions at 260–280 nm found in aromatic/polyaromatic compounds and conjugated molecules (including proteins and nucleic acids), respectively (Bikova and Treimanis 2004; Jia et al. 2007; Trabelsi et al. 2009; Rtimi et al. 2015).

In a previous study performed by our group (Tziourrou et al. 2020), during field experiments exposing similar plastic strips to the marine environment, it was found that the polymer type is only important for the colonization during the first period. PET is colonized faster during this initial phase than LDPE due to its low hydrophobicity. After this initial colonization has covered the surface, the next phase is similar for both polymers since the surface is at this point covered by similar molecules. This is not totally true for the present study, since the number of organisms in the bioreactors is not infinite and it is regulated by the addition of glucose. Although, the above microorganisms are attached to the surface only in some cases, biofilm formation is observed as indicated by peaks at the visible wavelength region.

In the present study, for LDPE, peaks indicating the presence of biofilm could be observed in the visible region for R2 and for PET, freshwater algae (R3) biofilm was also visible. PET in R3 is also the most densely populated sample both under the optical microscope (Fig. 2) and SEM (Fig. 5). Based on the DR spectra, different visible peaks for LDPE and PET were observed but, in both cases, the visible region peaks correspond to the peaks found in the water samples of the bioreactors (Fig. 6). This indicates that the growth of microorganisms depends not only on the length of contact time, but also on the type of material. Also, the patterns that can be seen for the fluctuation of peaks and their intensities could indicate periodic attachment and detachment of the microorganisms and/or the biofilm (Trulear and Characklis 1982).

Small differences were observed between DRS measurements for the samples that were wet compared to those that were allowed to dry at room temperature for 2 weeks. The peaks found at the UV region remain even after drying the samples and some of them increase in intensity. These peaks correspond to sorbed microorganisms. Peaks at visible wavelength region decrease in intensity suggesting the shrinking of the biofilm and the degradation of its colour but they were still present at the same wavelength. Alupoaei and Garcia-Rubio (2004) observed that the distribution of intensities as a function of wavelength depends on the size, shape, and optical properties of the microorganisms or cells. Another important point is the effect of relative humidity which varied with species (McEldowney and Fletcher 1988); for instance, *Pseudomonas* sp. increased under desiccation conditions.

### IR spectra interpretation

In both cases, different transmittance (%) values of the LDPE and PET samples were observed. This was likely related to biofilm characteristics. Specifically, according to Davis et al. (2011) the relative intensity among live and dead *Escherichia coli* cells were different using FT-IR spectra (700–4000 cm^−1^); lower intensity was observed for the dead cells.

**Fig. 13:**
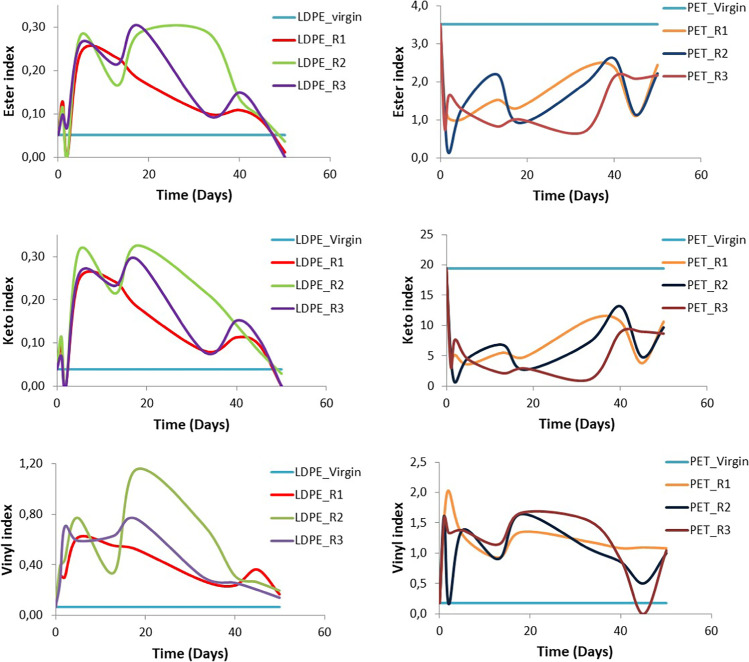
Ester (up), keto (middle), and vinyl (down) indices graphs of the LDPE (left) and PET (right) samples from day 1 to day 50. For the calculation of these indices, the peaks at 1740, 1715, and 1640 cm^−1^ are divided with the peak at 1465 cm^−1^ that corresponds to the invariant absorbance of the -CH_2_- bond to calculate the ester, keto, and vinyl indices, respectively (Kaberi et al. 2013; Fotopoulou and Karapanagioti 2015b; Tziourrou et al. 2019)

Based on the IR spectra, different fluctuations of ester, keto, and vinyl indices were observed (Fig. 13) from day 0 to day 50. Τhe virgin PET sample shows higher values in all the indices than the virgin LDPE sample as expected based on the carbonyl and aromatic groups present in PET monomer; [(virgin LDPE: ester in.= 0.051, keto in.= 0.039, vinyl in.= 0.067 similar indices values were calculated also by Tziourrou et al. (2021)), (virgin PET: ester in.= 3.5, keto in.= 19, vinyl in.= 0.18)]. This suggests that virgin PET surface is hydrophilic as expected.

For all the LDPE samples, all the indices demonstrated higher values (especially for R2) than the virgin LDPE. On the other hand, ester and keto indices for PET samples demonstrated lower values than virgin PET while vinyl index values seem to be similar with LDPE. The fluctuations of the values depend on the microorganisms (Albertsson et al. 1987). The patterns that can be seen for the indices could indicate periodic attachment and detachment of the microorganisms and/or the biofilm (Trulear and Characklis 1982). Actually, the indices increase and decrease several times, and actually more times for PET compared to LDPE corroborating previous findings the colonization of PET is faster than of LDPE (Tziourrou et al. 2020) due to its hydrophilicity.

Fotopoulou and Karapanagioti (2015b) suggested the colonization of PET by non-polar organisms assuming that the initial step for PET degradation is the colonization by these organisms. This is corroborated by the lower ester and keto indices of the PET covered by biofilm which however are higher than for LDPE suggesting that they are not that high due to the biofilm but due to PET itself. PET ester and keto indices have decreased due to biofilm covering most of the original carbonyl groups. The carbonyl groups formed due to biofilm are negligible compared to original PET carbonyl groups.

It is obvious that if only IR spectra are considered, the fluctuations observed in the DRS technique at the UV wavelength region is also identified. However, the difference on the surface of HDPE in R2 or PET in R3 cannot be identified. DRS technique was able to identify the formation of the biofilm both on wet and dry samples.

### Implications for plastic pollution

In this study, the first objective was to compare the biofilm formation on two commonly used plastics such as LDPE and PET. Sfaelou et al. (2015a) concluded that the polarity of the surface can influence biofilm quantity; more specifically, the surface with polar groups is more supportive to the formation of biofilm than a hydrophobic surface. Compared to hydrophobic surfaces, hydrophilic surfaces more quickly build up biomolecules that increase surface roughness, which may assist microbial adhesion (Bhagwat et al. 2021).

Indeed, plastic substrate properties affect or select biofilm communities to be initially attached on the biomolecules structure however biofilm formation causes physicochemical properties of the plastic surface to somewhat converge over time (McGivney et al. 2020) as in Fig. 13. This is also observed in the present study as well as in the previous one (Tziourrou et al. 2020). So, if all plastics have a similar surface, similar organisms are more abundant, and this can explain the observation by Krause et al. (2020) and Shen et al. (2021) that lower biodiversity is recorded on plastic biofilms compared to the surrounding environment. Also, according to Shen et al. (2021) plastic biofilms have a preference for pathogenic bacteria which should be true for all plastics since ultimately, they grow biofilms with similar surface properties.

The second parameter to be studied was the effect of the different environments and the various organisms found in these environments on the biofilm formation considering that plastics can be found in many different aquatic environment settings [e.g., marine environment: Baltic Sea (McGivney et al. 2020) and abyssal seafloor (Krause et al. 2020), freshwater: Xuanwu Lake (Shen et al. 2021), wastewater (Sfaelou et al. 2015a)]. Bacteria and algae found in R1 and R3 have a negative charge and varying degrees of hydrophobicity on their surfaces (Pal and Lavanya 2022; Ozkan and Berberoglou 2013). Since these microorganisms have a negative charge, they are attracted to positively charged surfaces. Negatively charged bacteria repel negatively charged surfaces by electrostatic forces. Ozkan and Berberoglou (2013) observed higher negatively surface charge for the saltwater algae than freshwater. This can explain the higher affinity of freshwater algae in R3 for the PET plastic surface compared to the affinity of saltwater algae in R1. The presence of salt in R1 creates an environment with high ionic strength that decreases the surface charge of PET making it more hydrophobic (Jones et al. 1999) causing it to have a similar behavior with HDPE but slightly higher initial attachment.

For the activated sludge system in R2, in our previous study, we have observed that although it contains functional groups that are both positive and negative, around neutral pH, the surface of the microorganisms is also neutral (Sfaelou et al. 2015b). This property makes the activated sludge less hydrophilic, and thus, it is easier to be attached to hydrophobic surfaces or to create flocs. Thus, this corroborates the formation of biofilm in R2 only on the hydrophobic LDPE and not on PET (even though PET had a higher initial colonization that created an initial roughness).

In a wastewater treatment plant, LDPE that floats in the aeration tank will obtain a biofilm with higher mass than PET. However, PET will obtain a coverage of biomolecules that will make it more hydrophobic.

In natural aquatic environments, LDPE floats and this property does not favor biofilm formation since it will mainly attract any hydrophobic microorganisms that are left on the surface. Most of the bacteria that are hydrophobic are benthic or are creating flocs (Fattom and Shilo 1984). Thus, LDPE that is moved to the bottom of the aquatic system is expected to have higher biofilm formation than the floating LDPE due to the presence of hydrophobic microorganisms found there. PET sorbs most aquatic biomolecules while floating and in freshwater after the initial roughness is formed, it can attract microalgae. Based on our previous experience with field experiments (Tziourrou et al. 2020), both LDPE and PET will form biofilm in the open sea because of the higher biodiversity of the sea compared to experimental setups used in the present study. Nevertheless, biofilm formation on LDPE will be higher and faster in benthos whereas on PET will be higher in freshwater systems.

## Conclusions

Based on microscopy observations, different species of microorganisms on both plastic surfaces were observed. DR spectra indicate different fluctuations among plastic materials, between the aquatic environments and relative humidity conditions (wet vs dry samples). Biofilm formation on the plastic samples was dependent of the material and type of the aquatic environment due to different biodiversity and differences of the hydrophobicity of the organisms. Alterations of the hydrophobicity of LDPE and PET samples were observed, based on IR spectra which were independent of aquatic environments, the same was true for the DRS peaks at the UV wavelength region. However, IR could not describe the formation of biofilm that created peaks in the visible region of DRS.

We propose future investigations to concentrate to the study (1) of biofilm formation on control degraded plastic samples due to plastic litter in the environment affected by solar UV radiation and (2) the correlation of DRS with microbiological analyses; in addition, more chemical composition information on plastic litter can be investigated, by inactive microorganisms on plastic surfaces (e.g., when membranes of dead microorganisms are broken down). Metagenomic sequencing of the bioreactor biomass (biofilm) DNA is required to analyze the microbial community and provide more meaningful results in the scientific community that is not only interested in the surface chemistry changes. Employing a negative control (sterile test water) with a strain that does not produce biofilm could be used to allow a better comparison with the original community in the three environments used.

## Supplementary information

ESM 1: Figure S1: The three glass batch reactor systems were developed for the experiment purposes (photograph from the 1st day of the experiment). Figure S2: SEM image of LDPE surface was in R2 for 33 days (up). Photomicrographs and elemental analysis (“spectrum 6” area) of the sample (LDPE in R2 bioreactor with synthetic fresh water / activate sludge / 33^th^ day); the short arrow shows silicon (Si) which is element of the shell of diatoms (Dugdale and Wilkerson 2001). Figure S3: SEM images of LDPE surface was in R3 for 33 days. Figure S4: DR spectra of wet biofilm on LDPE from R1, R2 and R3. Figure S5: DR spectra of wet biofilm on PET from R1, R2 and R3. Figure S6: DR spectra of dry biofilm on LDPE from R1, R2 and R3. Figure S7: DR spectra of dry biofilm on PET from R1, R2 and R3. Table S1: The F(R) intensity and the wavelength (nm) for selected peaks for wet LDPE samples from R1, R2 and R3. Table S2: The F(R) intensity and the wavelength (nm) for selected peaks for wet PET samples from R1, R2 and R3. Figure S8: The graphs illustrate the occurrence frequency of peaks (wet samples). Table S3: The F(R) intensity and the wavelength (nm) for selected peaks for dry LDPE samples from R1, R2 and R3. Table S4: The F(R) intensity and the wavelength (nm) for selected peaks for dry PET samples from R1, R2 and R3. Figure S9: According to IR spectra 718, 1462, 2847 and 2915 cm^−1^ are the IR peaks for virgin LDPE while for IR peaks for virgin PET see Table S5. Figure S10:IR spectra of LDPE samples from the three bioreactors. Figure S11: IR spectra of PET samples from the three bioreactors. Table S5: The main of the extra IR peaks of the LDPE sample. Table S6:The table below shows the main peaks for virgin PET.

## Abbreviations

ATR-FTIR: attenuated total reflectance–Fourier transform infrared spectroscopy
DR: diffuse reflectance
DRS: diffuse reflectance spectra
HDPE: high-density polyethylene
IR: infrared
LDPE: light density polyethylene
PET: polyethylene terephthalate
R1: bioreactor with seawater
R2: bioreactor with activated sludge
R3: bioreactor with freshwater algae
SEM: scanning electron microscopy
UV-vis: ultraviolet–visible spectrometry
