# Supplementary material for: Diffuse reflectance spectroscopy (DRS) and infrared (IR) measurements for studying biofilm formation on common plastic litter polymer (LDPE and PET) surfaces in three different laboratory aquatic environments
Source: Environ Sci Pollut Res Int. 2023 Apr 28;30(25):67499–512. doi: 10.1007/s11356-023-27163-2 (PMC10203007; doi:10.1007/s11356-023-27163-2)
Supplement: Supplementary file 1 — Figure S1: The three glass batch reactor systems were developed for the experiment purposes (photograph from the 1st day of the experiment). Figure S2: SEM image of LDPE surface was in R2 for 33 days (up). Photomicrographs and elemental analysis (“spectrum 6” area) of the sample (LDPE in R2 bioreactor with synthetic fresh water / activate sludge / 33th day); the short arrow shows silicon (Si) which is element of the shell of diatoms (Dugdale and Wilkerson 2001). Figure S3: SEM images of LDPE surface was in R3 for 33 days. Figure S4: DR spectra of wet biofilm on LDPE from R1, R2 and R3. Figure S5: DR spectra of wet biofilm on PET from R1, R2 and R3. Figure S6: DR spectra of dry biofilm on LDPE from R1, R2 and R3. Figure S7: DR spectra of dry biofilm on PET from R1, R2 and R3. Table S1: The F(R) intensity and the wavelength (nm) for selected peaks for wet LDPE samples from R1, R2 and R3. Table S2: The F(R) intensity and the wavelength (nm) for selected peaks for wet PET samples from R1, R2 and R3. Figure S8: The graphs illustrate the occurrence frequency of peaks (wet samples). Table S3: The F(R) intensity and the wavelength (nm) for selected peaks for dry LDPE samples from R1, R2 and R3. Table S4: The F(R) intensity and the wavelength (nm) for selected peaks for dry PET samples from R1, R2 and R3. Figure S9: According to IR spectra 718, 1462, 2847 and 2915 cm−1 are the IR peaks for virgin LDPE while for IR peaks for virgin PET see Table S5. Figure S10:IR spectra of LDPE samples from the three bioreactors. Figure S11: IR spectra of PET samples from the three bioreactors. Table S5: The main of the extra IR peaks of the LDPE sample. Table S6:The table below shows the main peaks for virgin PET. [file 11356_2023_27163_MOESM1_ESM.docx]

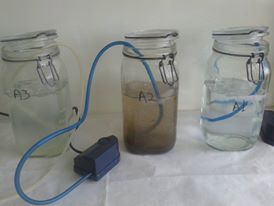


***Figure S1:*** *The three glass batch reactor systems were developed for the experiment purposes (photograph from the 1^st^ day of the experiment).*


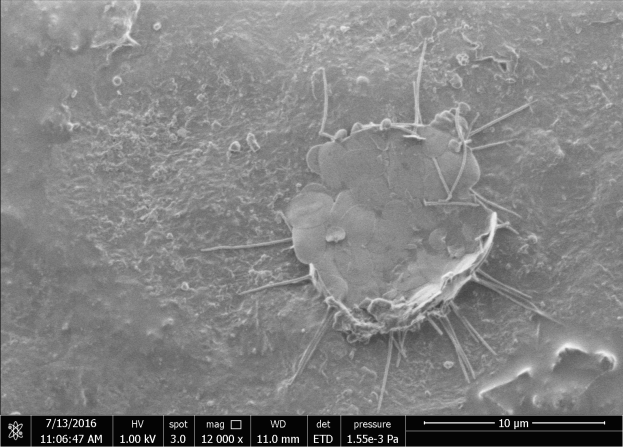


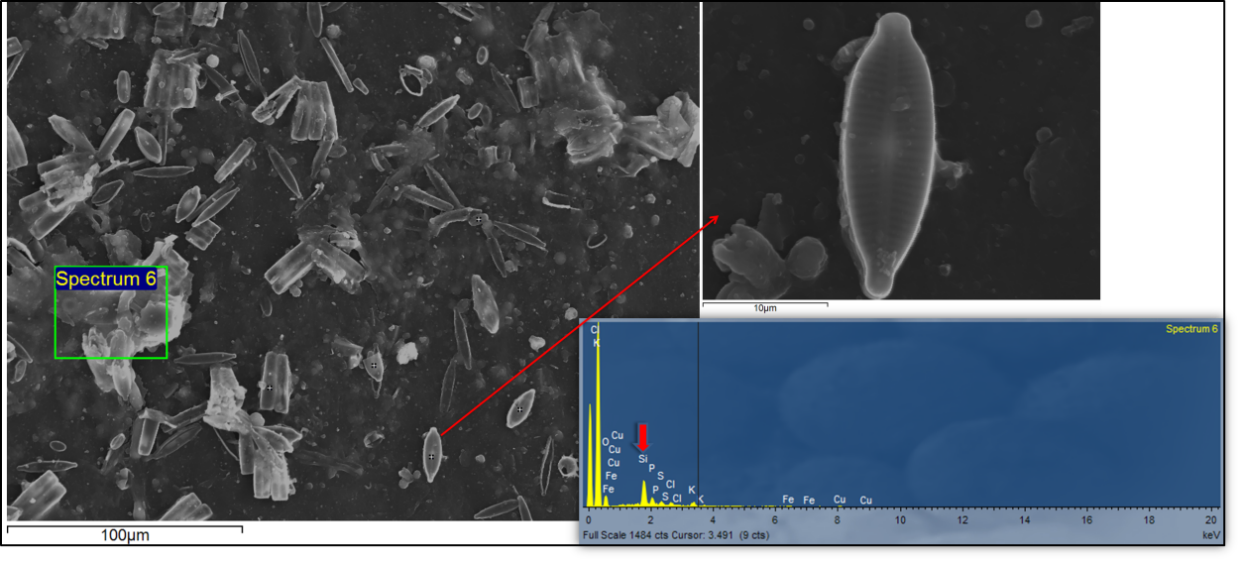


***Figure S2:*** *SEM image of LDPE surface was in R2 for 33 days (up). Photomicrographs and elemental analysis (“spectrum 6” area) of the sample (LDPE in R2 bioreactor with synthetic fresh water / activate sludge / 33^th^ day); the short arrow shows silicon (Si) which is element of the shell of diatoms (Dugdale and Wilkerson, 2001).*


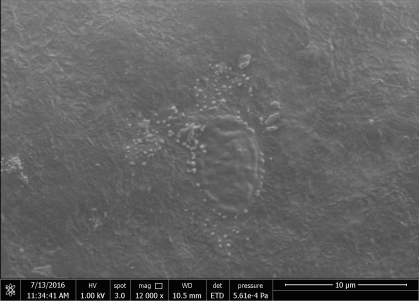

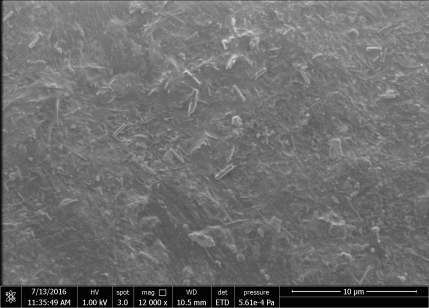


***Figure S3:*** *SEM images of LDPE surface was in R3 for 33 days.*

***Figure S4:*** *DR spectra of wet biofilm on LDPE from R1, R2 and R3.*

***Figure S5:*** *DR spectra of wet biofilm on PET from R1, R2 and R3.*

***Figure S6:*** *DR spectra of dry biofilm on LDPE from R1, R2 and R3.*

***Figure S7:*** *DR spectra of dry biofilm on PET from R1, R2 and R3*.

***Table S1:*** *The F(R) intensity and the wavelength (nm) for selected peaks for wet LDPE samples from R1, R2 and R3.*


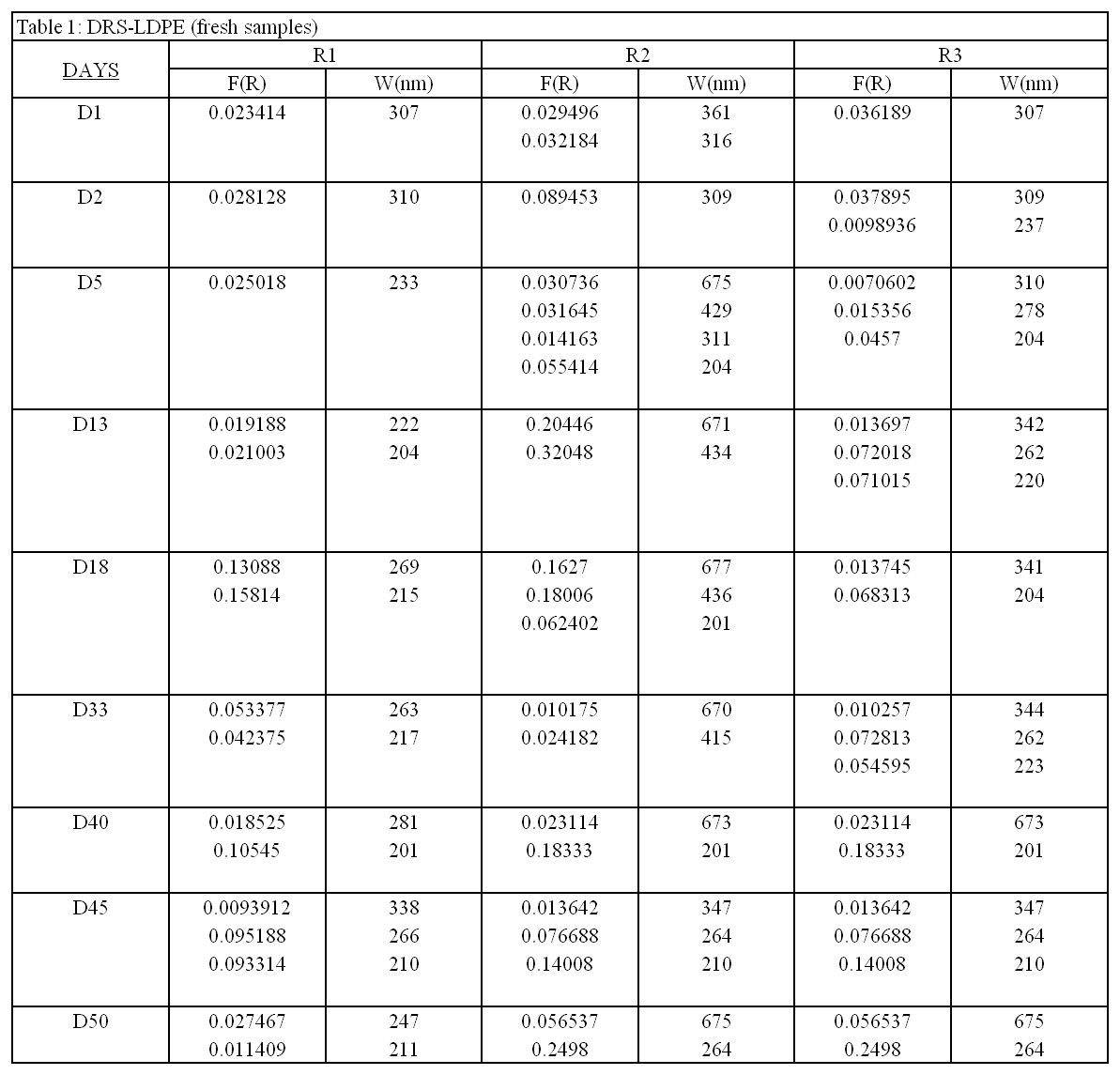


***Table S2:*** *The F(R) intensity and the wavelength (nm) for selected peaks for wet PET samples from R1, R2 and R3.*


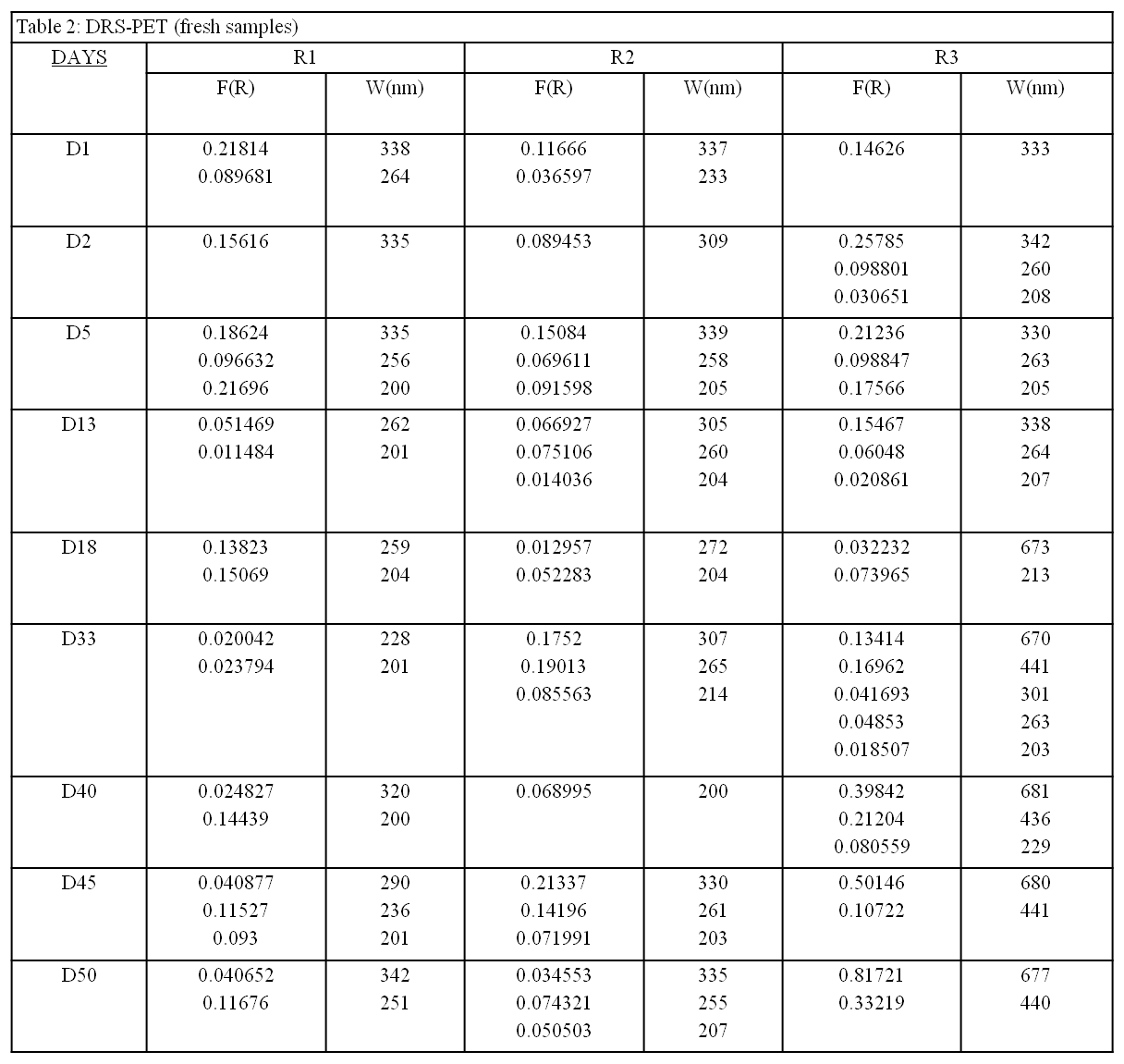


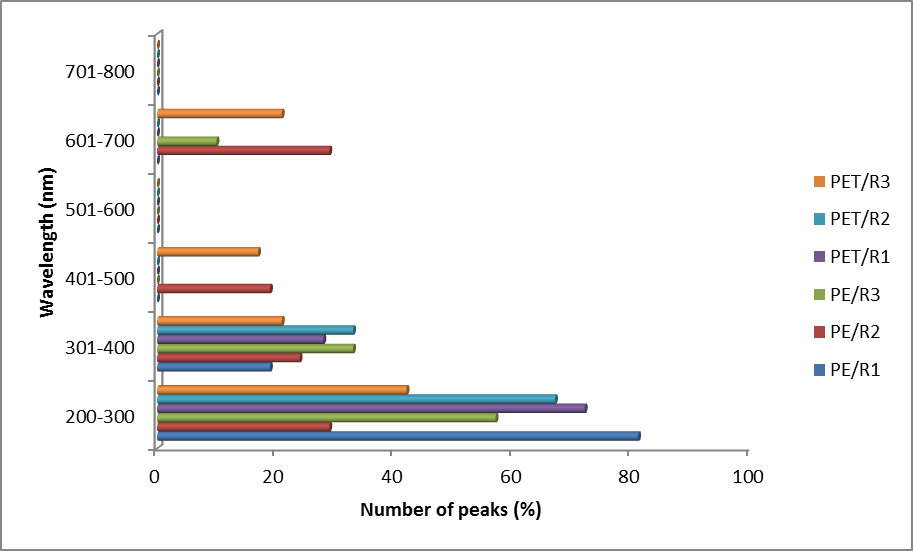


***Figure S8:*** *The graphs illustrate the occurrence frequency of peaks (wet samples).*

***Table S3:*** *The F(R) intensity and the wavelength (nm) for selected peaks for dry LDPE samples from R1, R2 and R3.*


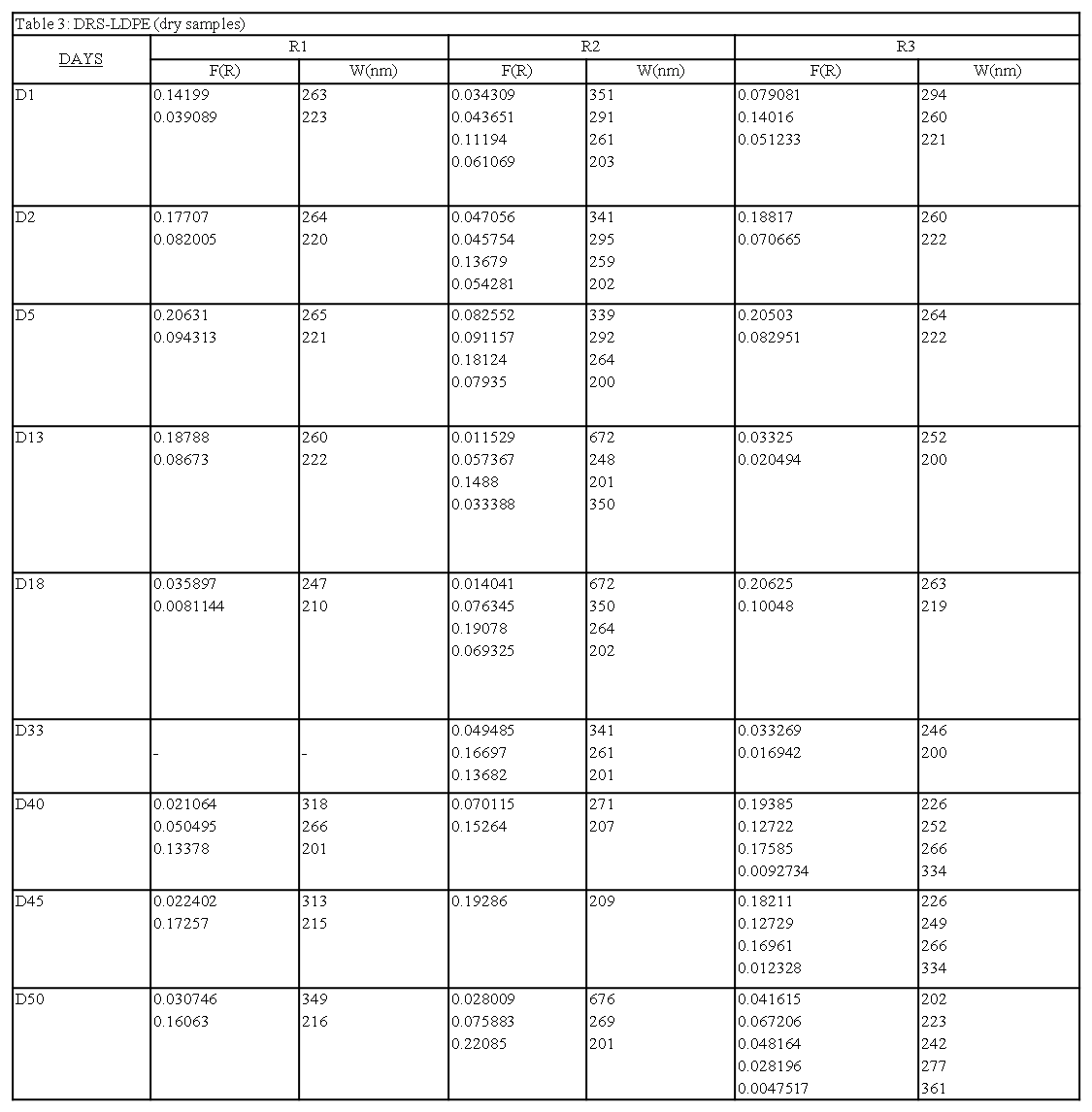


***Table S4:*** *The F(R) intensity and the wavelength (nm) for selected peaks for dry PET samples from R1, R2 and R3.*


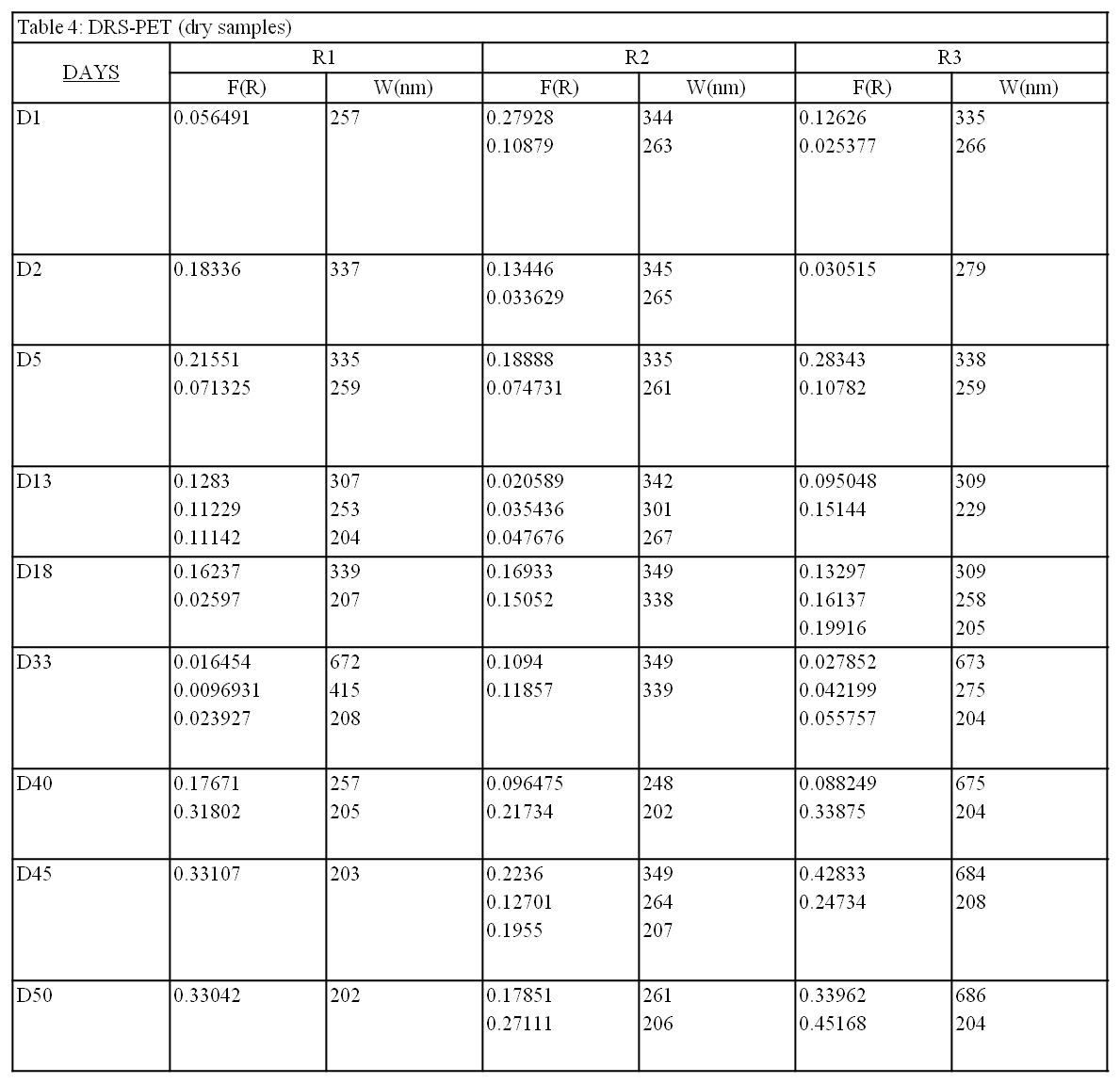


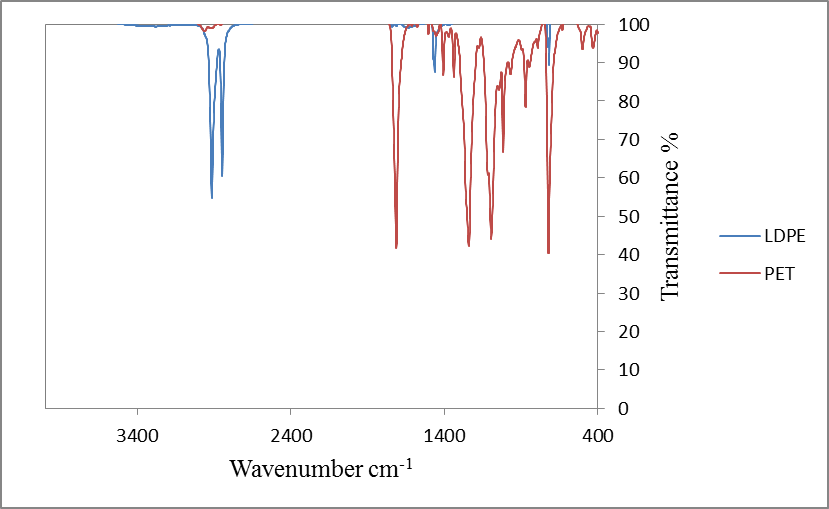


***Figure S9:*** *According to IR spectra* *718, 1462, 2847 and 2915 cm^−1^ are the IR peaks for virgin LDPE while for IR peaks for virgin PET see Table S5.*

***
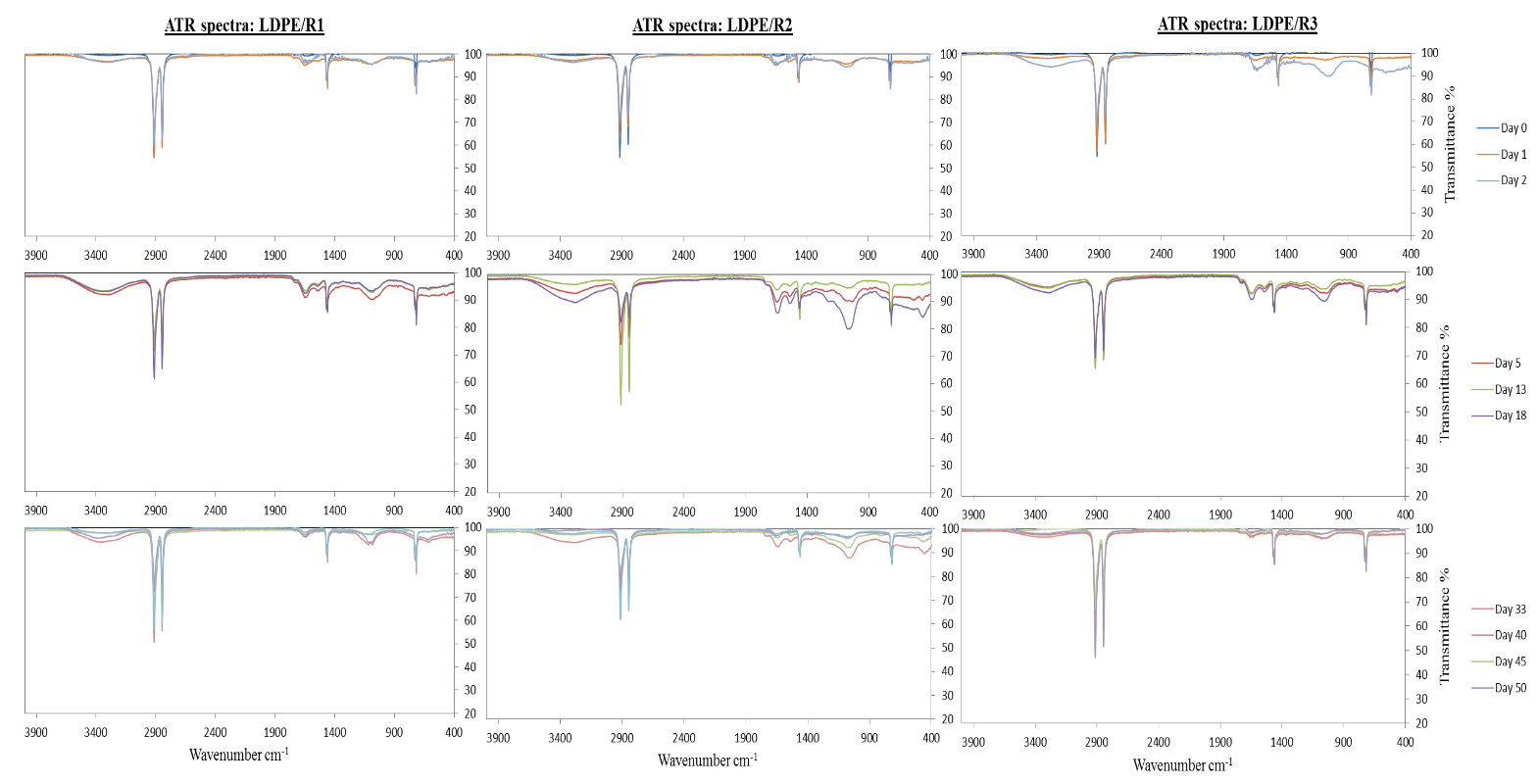
***

***Figure S10:****IR spectra of LDPE samples from the three bioreactors.*

***
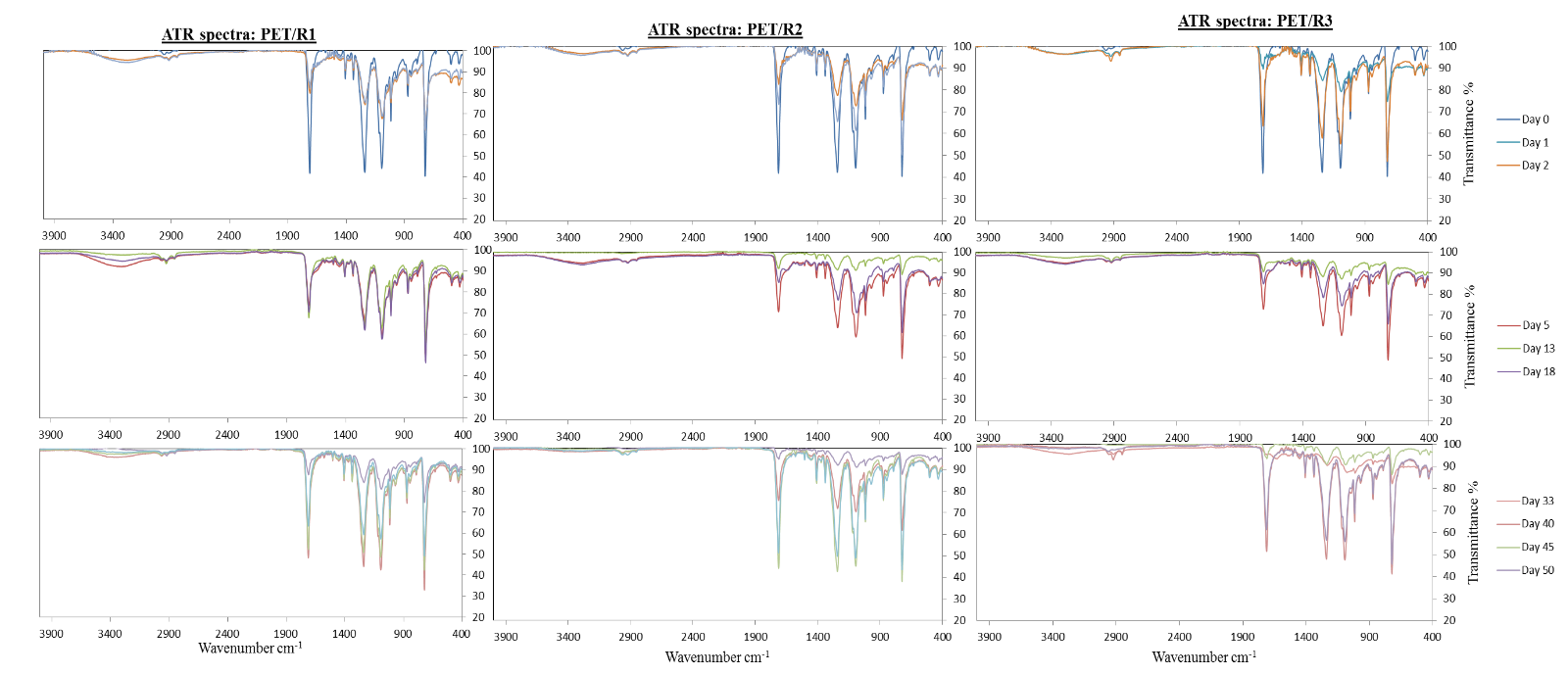
***

***Figure S11:*** *IR spectra of PET samples from the three bioreactors.*

***Table S5:*** *The main of the extra IR peaks of the LDPE sample.*

| Time  (Days) | **R1** | **R2** | **R3** |
| --- | --- | --- | --- |
|  | cm^-1^ | cm^-1^ | cm^-1^ |
| D1 | 1644**^1^** |  |  |
| D2 | 1642**^1^** |  | 1631**^1^**  1054**^2^** |
| D5 | 1642**^1^**  1081**^4^** | 1650**^1^**  1546**^7^**  1036**^2^** | 1638**^1^**  1538**^7^**  1054**^2^** |
| D13 | 1642**^1^**  1081**^4^** | 1648**^1^**  1544**^7^**  1064**^3^** | 1650**^1^**  1546**^7^**  1052**^2^** |
| D18 | 1642**^1^**  1081**^4^** | 1642**^1^**  1548**^7^**  1060**^3^** | 1650**^1^**  1542**^7^**  1050**^2^** |
| D33 | 1644**^1^**  1085**^5^** | 1638**^1^**  1066**^3^** |  |
| D40 |  | 1066**^3^** |  |
| D45 | 1654**^1^**  1099**^6^** |  |  |
| D50 | 1664**^8^** |  |  |
| **References**  **1. a)**Albertsson et al., 1987; Fotopoulou & Karapanagioti, 2015: **1640 cm^-1^ (**vinyl bond), **b)** Gajendiran et al., 2016: **1639 cm^-1^** (C-C=C symmetric of aromatic ring), **c)** Harshvardhan and Jha, 2013: **1650 cm^-1^** (double (vinyl) bond)**.**  **2. a)** Fotopoulou & Karapanagioti, 2012: “**1040 cm^-1^** is ester linkage”, **b)** Devlin et al., 2019**: 1020 – 1050 cm^-1^** (S=O), Parthasarathi et al., 2010: **1050 cm^-1^**.  **3.** Queiroz et al., 2015: **1066 cm^-1^** (C-O), Frost et al., 1998; Guerrero and Maier, 2018: **1066 cm^-1^**.  **4.** Gajendiran et al., 2016: **1078 cm^-1^** (C-O stretching of ether group).  **5.** Kazarian and Chan, 2013: **1085 cm^-1^ (**assigned to DNA phosphate backbone)**,** Prasad et al., 2011: **1085 cm^-1^**.  **6.** Jung et al., 2018: **1094 cm^-1^** C-O stretch.  **7.** Kovács et al., 2021:  **1542 cm^-1^** (NH_2_ and NH deformations), Riaz et al., 2018: **1544 cm^-1^**.  **8.** Rameshkumarr et al., 2021: **1664 cm^-1^** (presence of alkanes), Riaz et al., 2018: **1664 cm^-1^**. | | | |

***Table S6:****The table below shows the main peaks for virgin PET.*

| cm^−1^ | Reference |
| --- | --- |
| *Strong peaks* |  |
| 1713 | Ioakeimidis et al.,2016:  1715 cm^−1^ ketones (C = O) |
| 1244 | Ioakeimidis et al.,2016:  1245 cm^−1^ ether aromatic (C-O) |
| 1097 | Ioakeimidis et al.,2016:  1100 cm^−1^ ether aliphatic (C-O) |
| 724 | Ioakeimidis et al.,2016:  730 cm^−1^aromatic (C-H) |
| *Medium peaks* |  |
| 1407 | Mahal, 2021: 1407 cm^−1^ aromatic (C=C) |
| 1338 | Dubelley et al., 2017: 1340 cm^−1^ (CH_2_) |
| 1017 | Mahal, 2021: 1016 cm^−1^ bending mode of vibration in plane of benzene ring (C-H) |
| 871 | Ioakeimidis et al.,2016:  870 cm^−1^aromatic (C-H) |

**References**

Albertsson, A-C., Andersson, S. O.and Karlsson, S. 1987. The Mechanism of Biodegradation of Polyethylene. Polymer Degradation andStability 18: 73 – 87.

Devlin, A., Mauri, L., Guerrini, M., Yates, E. A., and Skidmore, M. A. 2019. The use of ATR-FTIR spectroscopy to characterise crude heparin samples by composition and structural features.  <https://doi.org/10.1101/744532>

Dubelley, F., Planes, E., Bas, C., Pons, E., Yrieix, B. and Flandin, L. 2017. The hygrothermal degradation of PET in laminated multilayer. European Polymer Journal. 87: 1 – 13.

Fotopoulou, K. N. andKarapanagioti, H. K. 2015. Surface properties of beached plastics.Environmental Science and Pollution Research. 22(14): 11022 – 11032.

Fotopoulou, K. N. andKarapanagioti, H.K., 2012. Surface properties of beached plastic pellets.Marine Environmental Research. 81: 70 – 77.

Frost, R. L., Kristof, J., Paroz, G. N. and Kloprogge, J. T. 1998. Molecular Structure of Dimethyl Sulfoxide Intercalated Kaolinites. The Journal of Physical ChemistryB.102: 8519 – 8532.

Gajendiran, A., Krishnamoorthy, S. and Abraham, J. 2016. Microbial degradation of low-density polyethylene (LDPE) by *Aspergillus clavatus* strain JASK1 isolated from landfill soil. 3 Biotech. 6: 52.

Guerrero, A. C. B. and Maier, M. S. 2018. Analysis of pictorial materials by Attenuated TotalReflectance Fourier Transform Infrared Spectroscopy. Cadernos do Lepaarq. XV(30): 267 – 276.

Harshvardhan, K. and Jha, B. 2013. Biodegradation of low-density polyethylene by marine bacteria frompelagic waters, Arabian Sea, India. Marine Pollution Bulletin. 77: 100 – 106.

Ioakeimidis, C., Fotopoulou, K. N., Karapanagioti, H. K. Geraga, M, Zeri, C., Papathanassiou, E., Galgani, F. and Papatheodorou, G. 2016. The degradation potential of PET bottles in the marine environment: An ATR-FTIR based approach. Scientific Reports. 6: 23501.

Jung, M. R., Horgen, F. D., Orski, S.V., Rodriguez, C.V., Beers, K.L., Balazs, G. H., Todd Jones, T., Work, T.M., Brignac, K. C., Royer, S.-J., Hyrenbach, K.D., Jensen, B. A. and Lynch, J.M., 2018. Validation of ATR FT-IR to identify polymers of plastic marine debris, including those ingested by marine organism. Marine Pollution Bulletin. 127: 704 – 716.

Kazarian, S. G. and Chan, K. L. A. 2013. ATR-FTIR spectroscopic imaging: recent advances and applications to biological systems. Analyst. 138: 1940 – 1951. DOI: 10.1039/c3an36865c

Kovács, R. L., Csontos, M., Gyöngyösi, S., Elek, J., Parditka, B., Deák, G., Kuki, À., Kѐki, S., Erdѐlyi, Z. 2021. Surface characterization of plasma-modified low density polyethylene by attenuated total reflectance fourier-transform infrared (ATR-FTIR) spectroscopy combined with chemometrics. Polymer Testing. 96: 107080.

Mahal, Z. 2021. Enhanced Hydrolysis of Polyethylene Terephthalate (PET) plastics by Ozone and Ultrasound Pretreatment. Thesis. Master of Science in Biology. Lakehead University. <https://knowledgecommons.lakeheadu.ca/handle/2453/4811>

Parthasarathi, V., Sundaresan, B., Dhanalakshmi, V. and Anbarasan, R. 2010. Functionalization of HDPE with aminoester and hydroxyester by thermolysis method – An FTIR-RI approach. Thermochimica Acta. 510: 61 – 67.

Prasad, S. G., De, A. and De, U. 2011. Structural and Optical Investigations of Radiation Damage in Transparent PET Polymer Films. International Journal of Spectroscopy. Article ID 810936.

Queiroz, M. F., Melo, K. R. T., Sabry, D. A., Sassaki, G. L. and Rocha, H. A. O . 2015. Does the Use of Chitosan Contribute to Oxalate Kidney Stone Formation? Marine Drugs. 13: 141 – 158.

Rameshkumarr, C., Anderson, A. and Ravichandran, S. 2021. UV, FIRT and surface properties of fiber reinforced low-density polyethylene laminated composites. Materials Today: Proceedings. 47(7): 6216 – 6223.

Riaz, M, Yan, L., Wu X. W., Hussain S, Aziz O, Wang, Y. H., Imran M, Jiang C. C. 2018. Boron alleviates the aluminum toxicity in trifoliate orange by regulating antioxidant defense system and reducing root cell injury. J Environ Manag. 208: 149 – 158.

Riaza, T., Zeeshana, R., Zarifa, F., Ilyasa, K., Muhammada, N., Safia, S. Z., Rahima, A., Rizvib, S. A. A. and Rehman, I. U. 2018. FTIR analysis of natural and synthetic collagen. Applied Spectroscopy Reviews. 53(9): 703 – 475.
